# Adipose Stem Cell Translational Applications: From Bench-to-Bedside

**DOI:** 10.3390/ijms19113475

**Published:** 2018-11-05

**Authors:** Chiara Argentati, Francesco Morena, Martina Bazzucchi, Ilaria Armentano, Carla Emiliani, Sabata Martino

**Affiliations:** 1 Department of Chemistry, Biology and Biotechnologies, University of Perugia, Via del Giochetto, 06126 Perugia, Italy; chiara.argentati89@gmail.com (C.A.); effemorena@gmail.com (F.M.); marti89.b@libero.it (M.B.); carla.emiliani@unipg.it (C.E.); 2 Department of Ecological and Biological Sciences, Tuscia University Largo dell’Università, snc, 01100 Viterbo, Italy; ilaria.armentano@unitus.it; 3 CEMIN, Center of Excellence on Nanostructured Innovative Materials, Via del Giochetto, 06126 Perugia, Italy

**Keywords:** regenerative medicine, gene/cell therapy, biomaterials, tissue engineering

## Abstract

During the last five years, there has been a significantly increasing interest in adult adipose stem cells (ASCs) as a suitable tool for translational medicine applications. The abundant and renewable source of ASCs and the relatively simple procedure for cell isolation are only some of the reasons for this success. Here, we document the advances in the biology and in the innovative biotechnological applications of ASCs. We discuss how the multipotential property boosts ASCs toward mesenchymal and non-mesenchymal differentiation cell lineages and how their character is maintained even if they are combined with gene delivery systems and/or biomaterials, both in vitro and in vivo.

## 1. Introduction

In this work, we conducted a thorough systematic review of the adult adipose stem cells (ASCs). We started with the discussion of the development origin and the stemness properties of ASCs and continued reporting the translating research, highlighting the applications of ASCs in transplantation, gene therapy, and in tissue engineering.

### The Stem Cell Paradigm

**Figure 1:**
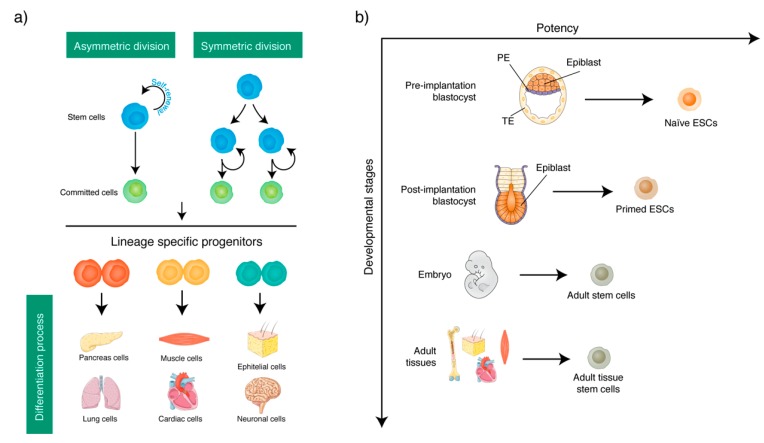
Paradigm of stem cells. (**a**) Asymmetric and symmetric division of stem cells give rise committed cells that generate lineage specific progenitors. The latter generate differentiated cells. (**b**) The cartoon shows the origin of the different stem cell types during the developmental stages.

The paradigm of stem cell biology is based on the stemness properties of self-renewal and pluri/multi-potency. By their self-renewal property, the stem cells proliferate to maintain the stem cell reservoir in the undifferentiated state throughout life (Figure 1a) [1,2]. By their pluri/multi-potency property stem cells give rise to different types of differentiated cells (Figure 1a) [2,3,4,5,6,7]. Both molecular events take place within the niche, a tissue area where stem cells reside. Here, the architecture is provided by the interaction of stem cells and support from neighboring cells, whereas the stem cell behavior and functions (e.g., proliferation, morphology, motility, adhesion, autocrine/paracrine activity, and gene expression) are guaranteed by spatially and temporally organized compositions of growth factors such as, cytokines, chemokines, transmembrane receptor ligands, and extracellular matrix (ECM) molecules [2,3,4,5,6,7,8].

Self-renewal and pluri/multipotency properties are the consequence of a particular cell division mechanism, called as asymmetric division, in which stem cells generate a daughter cell that remains a stem cell identical to the mother and another daughter cell with committed characteristics [9,10,11]. The latter, under selected signals, generates a progenitor cell that enters in selected differentiating programs. This gives rise to specialized tissue-specific cell lineages, and contributes to the maintenance of the tissue homeostasis (Figure 1a) [2,3,4,5,6,7]. In some cases, stem cells divide by the symmetric division mechanism and generate two daughter cells identical to the stem cell mother [12]. Both mechanisms maintain the stem cell number balancing quiescent and committed/progenitors cells within the niche [13].

It should be noted that while the self-renewal property remains unchanged in stem cells for their entire life, the lineage differentiation potentials are strictly associated with the development process (Figure 1b). Specifically: (i) Naïve- and primed-embryonic stem cells. These are stem cells from the early transition stages of embryo development that differ in epigenetic signatures. In fact, naïve and primed states have distinct signaling pathways and their transition is accompanied by a cascade of molecular events that govern the specification of trophectoderm and primitive endoderm lineages [14,15,16]. In the mouse, the naïve state corresponds to the embryo epiblast stage while the primed state corresponds to the embryo post-implantation stage [15,16,17]. Naïve and primed stem cells have different telomere lengthening, DNA methylation, and gene expression signature. Of note, although naïve and primed stem cells have pluripotency properties, only the naïve stem cells have the capacity of chimera formation. Finally, mouse naïve stem cells generate mESC lines whereas the primed stem cells generate mEpiSC lines in vitro [15,16,17,18].

In humans, the correlation of naïve and primed pluripotent state with the early stages of embryo development is still requires further investigation. This emerges by discrepancies in the literature do to the difficulty of recapitulating the developmental events in a very representative study model [15,16,17,18]. It has been confirmed that naïve stem cells come from the pre-implantation state and primed stem cells come from the post-implantation state; however, it has also been highlighted that the process of specification of trophectoderm and primitive endoderm lineages occurs almost at the pre-implantation embryo stage [18]. As described in mice and humans, naïve and primed differ in telomere lengthening, mechanisms of DNA repair, and of a broader repressive epigenetic signature such as DNA methylation. Finally, as-of-now, the contribution of the post-implantation epiblast of non-human primates to chimera formation remains elusive [15,16,17,18].

(ii) Adult stem cells or tissue stem cells. These stem cells are generated during the ontogeny and have self-renewal and multipotency properties. The more restrictive differentiation potentials should be accounted for by the characteristics of their embryonic layer of origin, which is maintained by the adult stem cells (Table 1) [7,19,20,21,22,23,24,25,26,27]. Adult stem cells persist within the niche of adult tissues and organs through life, replacing cells within the tissue under physiological and pathological conditions [7,19,20,21,22,23,24,25,26,27].

(iii) Cancer stem cells. These putative stem cells have been isolated from several cancers [11,28,29]. Currently, cancer stem cells have been recognized as cells that cause tumor progression and are considered as a target of cancer therapy. They have self-renewal and multipotency properties and others critical characteristics necessary for the metastatic development [11,28,29].

Starting in 2006, a new type of stem cells has emerged [30]. These stem cells which are called induced pluripotent stem cells (iPSCs) were first generated from terminally differentiated somatic cells engineered with four genes (*OCT4*, *SOX2*, *KLF4*, *cMYC*). These were capable of changing the epigenetic steady-state of the fibroblasts and activating the epigenetic stem cell programs as they acquire self-renewal and pluripotency capability [31,32,33,34]. Currently, iPSCs might be generated with different cocktails of genes [35] and from different types of somatic cells [36]. The greater advantage of iPSCs is for modeling human diseases in vitro and in turn for exploring the molecular basis of given disorders and developing effective therapeutic drugs as well [36]. Of note, recent studies have clearly demonstrated the variability of human iPSCs, as they have shown varied differentiating capacity toward specific lineages. Therefore, a current challenge is the definition of a more universal protocol of differentiation in order to ensure the reliability of large scale-up applications and effective personalized therapies [37].

## 2. Adipose Stem Cells

Adipose stem cells (ASCs) are adult stem cells with self-renewal and multipotency properties isolated from the adipose tissue [64,65,66,67,68]. The adipose tissue is a highly heterogeneous tissue (e.g., stem/precursor cells; different type of adipocytes (white, beige, brown), endothelial cells; and pericytes) either among individuals or within the same individual, if different sites of fat deposits are compared [41,69]. It has been shown that such donor-dependent differences could result in a different stem cell yield, proliferation, and differentiation capacity [67,68,70,71,72]. Of note, the high distribution of adipose tissue in the body and the relatively easy and safe method of tissue recovery allow the isolation of ASCs without morbidity for donors [40,41,69,73].

### 2.1. History of Adipose Stem Cells

The isolation of progenitor cells from adipose tissue was first described in rodents in 1964 by Rodbell [74,75]. In 1977, Van and Roncari analyzing the adipose tissue of adult rats reported the presence of cells with a proliferative rate and morphological characteristics similar to adipocytes [76]. In 1989 Hauner and co-authors, isolated and identified the preadipocytes as adipocyte precursor cells and studied the role of the glucocorticoids on their differentiation by using human subcutaneous abdominal deposit [77]. The introduction of liposuction as a surgical procedure for harvesting the adipose tissue has dramatically improved research in this area [78,79]. Thus, Zuk and co-authors isolated a cell population called as “fibroblast-like cells” or “processed lipoaspirate cells” with the characteristics of stable doubling-time proliferation, almost absence of senescence signs, and expression of mesenchymal markers, and these characteristics are maintained over a prolonged period of time in vitro [79,80]. Of particular note is that these cells also exhibited a multipotency property. In fact, in 2002, Mizuno et al. demonstrated the myogenic differentiation from human processed lipoaspirate cells of eight patients who underwent cosmetic surgery repair [81]. In the same year, Safford and colleagues explored the neurogenic differentiation of adipose-derived stromal cells, both in murine and a human in vivo model [82]. One year later, Gimble et al. isolated the adipose-derived adult stem cells from rodent lipoaspirate with a new protocol and confirmed their multipotency activities [83]. In 2004, Miranville and co-authors described the presence of adipose stem/progenitor cells within the stromal vascular fraction (SVF) of human adipose tissue obtained from different sources: subcutaneous gluteal fat, subcutaneous abdominal, and visceral abdominal [84]. From 2005, the surface markers of human adipose-derived stromal cells isolated from adult human adipose tissue were published [85,86]. Later studies focused on understanding whether adipose-derived stromal cells originated from adipose tissue were mesenchymal cells or peripheral blood stem cells that pass through fat tissue, as adipose tissue, similar to the bone marrow, has a mesodermal origin [87]. Within this aim, comparative studies, conducted in mesenchymal stem cells isolated from adipose tissue and from bone marrow, revealed the presence of some common mesenchymal surface markers but a different degree of differentiation toward the different cell lineages [88,89]. More recently, differences on the phenotypic markers were also identified [90].

In agreement with a consensus of participants to the second annual meeting of the Society of Applied Fat Technology (3–5 October 2004, Pittsburgh, PA, USA), the research community will refer to all types of adipose-derived stem cells as adipose stem cells (ASCs), from now on.

### 2.2. Isolation Methods of Human Adipose Stem Cells

**Figure 2:**
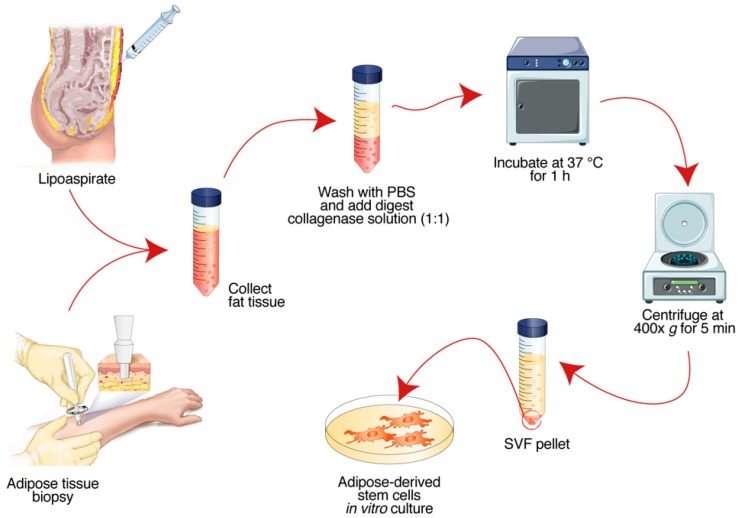
The figure shows the main steps for generating ASCs from lipoaspirate or subcutaneous adipose tissue (see the text for details).

Adipose tissue can be easily obtained by adipose tissue biopsy or lipoaspirate (Figure 2). In both cases, the adipose tissue samples can be stored at 4 °C for no longer than 24 h before use [91]. In the case of biopsy, the adipose tissue is crushed by hand into a Petri dish to obtain small fragments. In the case of lipoaspirate, the procedure is highly simplified due to the finely ground fat fragmentation generated with the size of the cannula used in the liposuction [91]. Fat fragments from lipoaspirate or biopsy are digested by incubation at 37 °C with collagenase. After centrifugation, the cell pellet is resuspended in the culture medium. These procedures should be improved [92,93,94]. For instance, Rada et al. have described a new standard method for ASCs isolation from lipoaspirate through antibody-coated immuno-magnetic spheres [92]. Independent studies have shown that lipoaspirate or fat biopsy gives rise adherent stromal cells with adipocyte progenitors characteristics [93,94]. In this regard, our recent studies on ASCs isolation and characterization have shown that stem cells obtained from breast biopsy have a lower differentiation rate than lipoaspirate ASCs. Following induction of osteogenic differentiation under inducers molecules (by SingleQuots (Lonza Walkersville, Inc., Walkersville, MD, USA): dexamethasone, l-glutamine, ascorbate, pen/strep, mesenchymal cell growth supplement (MCGS), and b-glycerophosphate), it was demonstrated that the ASCs isolated from lipoaspirate respond better to osteogenic differentiation rather than the ASCs obtained from breast tissue [73].

In the most common method of culture, ASCs are seeded onto the culture flasks and incubated at 37 °C, 5% CO_2_ in growth medium containing 10% Fetal Bovine Serum, 1% penicillin and streptomycin, and 1 mM Glutamine [64,73]. Recent results have demonstrated that ASCs may be expanded in culture for almost 70 doubling times, much longer than other types of mesenchymal stem cells such as those obtained from bone marrow [95].

To our knowledge, few studies have explored the effect of cryopreservation procedures on human ASCs. Available data indicated that hASCs that underwent multiple rounds of cryopreservation, or thawed for a long-term period, maintained the stemness properties and in general viability, gene expression, immunophenotype markers, and differentiation capacity without alteration [96,97,98].

These data represent a good basis for biobanking ASCs [99]. This procedure allows accomplishing an archive of stem cells avoiding donor variables such as health status and age at the time of collection. With the same aim, the research attempt to develop safe and efficient long-term biobanks for human fat to be use as source for ASCs [100].

### 2.3. Property of Multipotency and Self-Renewal of Adipose Stem Cells

The ASCs are adult stem cells with a mesodermal origin (Table 1) [40,41,80] as they express mesenchymal phenotypic markers (CD73, CD90, CD105) whereas they are negative for CD34, CD45, and HLA-DR markers [65]. ASCs have self-renewal property as demonstrated by the Colon Forming Unit formation (CFU-F) assay [39,40,41,66]. They have multipotency property, as they have the capability to differentiate toward different mesenchymal cell lineages [39,65,101,102,103,104,105,106,107,108,109]. Interestingly, ASCs also have a higher responsiveness to inducers of non-mesenchymal cell lineages differentiation [39,65,101,102,103,104,105,106,107,108,109].

#### 2.3.1. Mesenchymal Cell Lineages

**Adipogenic**. Given the origin of the ASCs it is not surprising that, when grown in adipogenic medium (containing: rh-insulin, l-glutamine, MCGS, dexamethasone, indomethacin, 3-isobuty-lmethylxanthine, penicillin/streptomycin), ASCs express different adipocyte genes including leptin, lipoprotein lipase, peroxisome proliferator-activated receptor gamma 2 (PPARγ2), Glut4 and develop intracellular vacuoles loaded with lipids [39,41,73]. Furthermore, the capability of these cells to differentiate in vivo towards cells of the adipocytic lineage has also been maintained even after implantation of stem cells in vivo [39,110].

**Osteogenic**. The osteogenic differentiation of ASCs, may be achieved by using inducing osteogenic factors (e.g., dexamethasone, ascorbic acid/ascorbate 2-phosphate, and β-glycerophosphates, vitamin D3, transforming growth factor-β and bone morphogenetic proteins), in growth culture medium. As a consequence ASCs express early osteogenic markers (alkaline phosphatase and BMPII) and later osteogenic markers (osteocalcin, osteogenic transcription factor Runx2, osteonectin, osteopontin, bone morphogenic protein-2, and Osterix) [41,73,101,111].

**Chondrogenic**. The chondrogenic differentiation of ASCs may be obtained by using specifically selected inducer molecules, such as: transforming growth factors β 1 and 3 (TGF-β 1, TGF-β 3), bone morphogenetic protein 4 (BMP 4), basic fibroblast growth factor (bFGF). These factors are supplemented to the growth culture medium of ASCs and might be used alone or in combination. However, other differentiation protocols used as a chondrogenic inducer are ascorbate-2-phosphate, TGF-β 1, and insulin [112,113]. The chondrogenic differentiation was demonstrated by the presence of a high concentration of glycosaminoglycans (GAGs) and by the presence of sulfate GAGs [112,113].

#### 2.3.2. Non-Mesenchymal Lineage

It should be noted that ASCs also have the capability to differentiate toward non-mesenchymal cell lineages such as myogenic, neuronal and endothelial [104,105,106,107,108,109].

**Skeletal myogenic differentiation**. The first evidence of myogenic differentiation was observed in ASCs cultured in myogenic medium (control medium (Dulbecco modified Eagle medium, 10% fetal bovine serum, 1% antibiotic/antimycotic) supplemented with 5% horse serum and 50 µm hydrocortisone) for 6 weeks, as demonstrated by the expression of muscle-specific transcription factor MyoD1 and the myosin heavy chain [81]. This leads to changes in the morphological characteristics of cells that become multinucleated and elongated with myofibrillar structures that appeared after two weeks in culture. Bacau and co-authors demonstrated myogenic potentials in vivo, transplanting ASCs in an injured rabbit muscle. They observed an increase in muscle weight, a restoration of the fiber cross section area together with the contractile force compared to the damaged control [72,104,114].

**Cardiac**. ASCs have been explored for the capability to differentiate to cardiomyocytes in vitro [41]. Interesting results were those obtained from the study by Planat-Bénard and co-authors which showed how freshly extracted ASCs, put into a semi-solid culture, spontaneously gave rise to beating cells [115]. Further studies addressing the capability of ASCs to regenerate damaged myocardium in vivo are also ongoing [19,116,117].

**Neurogenic**. The differentiation of ASCs in vitro toward the neural lineage has also been explored [105,106]. For instance, the treatment of human or rat ASCs with beta-mercaptoethanol results in a rapid transition of cells to neuronal morphology and expression of neuronal markers such as nestin expression, neuron-specific enolase, and neuron protein [82]. The same results were observed by exposure of ASCs to isobutylmethylxanthine and dibutyryl cAMP or forskolin and butylated hydroxyanisole [118]. In vivo data on the neural potential of ASCs, currently, are limited but promising [106,119].

**Endothelial**. Generation of endothelial cells from ASCs has been addressed [107,108,109,120]. For instance, Lee et al. demonstrated that treatment of ASCs with endothelin-1 (a paracrine factor secreted by endothelial cells), caused their differentiation toward endothelial cells [107]. ASCs incubated in conditioned medium, obtained by culture of human umbilical vein endothelial cells expressed high levels of vascular endothelial growth factor and placenta growth factor. They also showed a high proliferation rate, invasion and angiogenesis capability. Therefore ASCs, are evaluated as a potential treatment for preeclampsia, a hypertensive complication in pregnancy that involves endothelial dysfunction [108].

## 3. Adipose Stem Cells and Regenerative Medicine

Regenerative medicine aims at creating functional tissues by repairing or replacing worn out/damaged tissues or organs due to genetic, trauma, ageing, and degenerative defects [4,121,122,123]. The rationale is based either on the therapeutic potential of stem cells to replace damaged or dead cells with the newly differentiated progenies in order to repopulate the tissue [122,123], or on their capability to actively contribute to the tissue repair and regeneration by autocrine and paracrine actions through the secretion of growth factors, cytokines, and ECM molecules [124,125,126].

**Figure 3:**
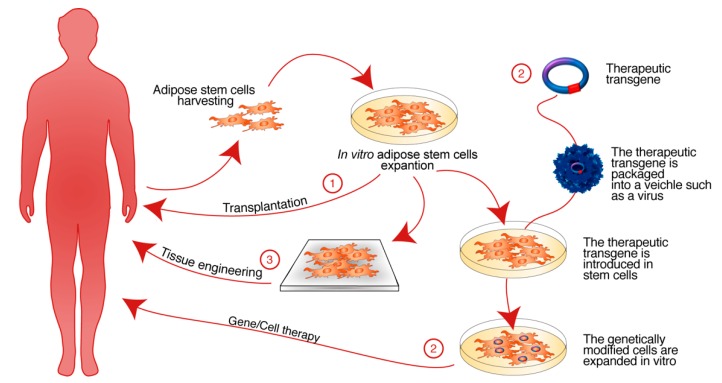
Regenerative medicine paradigm. The cartoon schematizes the three current strategies of regenerative medicine: (1) Transplantation. Autologous ASCs are expanded in vitro and are implanted in the host recipient tissues directly (see Section 3.1). (2) Gene/Cell therapy. Autologous ASCs are expanded in vitro and are transduced with viral-vector carrying the therapeutic gene and then are implanted in the host recipient tissues (see Section 3.2). (3) Tissue Engineering. Autologous ASCs are expanded in vitro and are combined with biomaterials, then are implanted in the recipient host to regenerate damaged tissues (see Section 3.3).

The overall biological functions are maintained by stem cells even if they are transplanted in vivo into a host tissue [7,19,23,48,127,128], or if they are combined with a therapeutic gene or with a biomaterial to generate an ex vivo tissue (Figure 3) [40,73,127,128,129,130,131,132,133,134,135,136,137,138,139]. For clinical applications, stem cells should meet some criteria: (i) they must be in abundant quantities; (ii) they must be obtained with a minimum invasive procedure for the patient; (iii) they should be able to differentiate toward multiple cell-derived pathways in a reproducible manner; and (iv) they must be safe after transplantation into an autologous or allogeneic recipient host [41,140,141]. In this regard, ASCs represent a suitable source for the treatment of a large number of diseases [40,140,142] (Table 2).

### 3.1. Modulation of the Cell/Tissue Milieu Capability of ASCs

It is a general concern that the therapeutic potential of stem cells transplanted in a host tissue may be mostly due to their paracrine actions instead of the cell replacement and differentiation alone [143,144,145,146]. In fact, stem cells secrete a panel of trophic factors, such as cytokines, growth factors, chemokines in the microenvironment to control cell proliferation, migration and differentiation, and to provide cytoprotection [124,125]. Additional paracrine molecules released by stem cells include: (i) antioxidants and anti-apoptotic molecules to protect cells from oxygen free radicals, (ii) angiogenic factors, (iii) factors controlling the ECM homeostasis, and (iv) anti-inflammatory or immunosuppressive factors [124,125,126,145]. ASCs have a high paracrine activity either in physiological condition or in regenerative medicine applications [125]. A recent study showed that high levels of angiogenic factors (Hepatocyte Growth Factor, Vascular-Endothelial Growth Factor, and basic Fibroblast Growth Factor) secreted by ASC sheets on rat myocardial infarction under normoxic and hypoxic conditions, reduced the cell apoptosis and improved cardiac function. Interestingly, the ASC sheets were more effective than myoblast cell sheets [147]. Many reports have documented the immunomodulatory activity of ASCs. It was demonstrated that ASCs may alleviate sepsis inducing a phenotype modification in monocytes by the reduction of the Tumor Necrosis Factor-α and increased the Interleukin-10 expression. The mechanism included the increase of levels of the other immunomodulatory molecules Prostaglandin-E2, Cyclooxygenase-2, and Prostaglandin-EP4 [148]. Additionally, Bahrami and co-authors demonstrated that the production of immunomodulatory molecules Indoleamine 2,3-dioxigenase 1, Indoleamine 2,3-dioxigenase 2 and human leukocyte antigen-G molecules were higher in ASCs isolated from breast cancer patients (levels were in the order stage III tumors > stage II) than those from healthy subjects [149]. Interestingly, ASCs seem to have a more marked effect on dendritic cell differentiation than Bone Marrow-MSCs thus confirming the difference between the two types of stem cells [150,151].

Finally numerous preclinical studies demonstrated that the paracrine activity of ASCs improve bone healing [125,146].

### 3.2. Adipose Stem Cell Transplantation

The adipose tissue has offered a great promise over the years for reconstructive surgery and its therapeutic use in pre-clinical studies and clinical trials have been well documented (Figure 3; Table 2) [68,140,152]. ASCs and free fat have been used clinically for the repair of soft tissue such as breast, face defects, and pathological disorders as lipodystrophy [153,154]. ASCs were also used for orthopedic applications, treatment of inflammatory diseases (such as the fistula induced by Crohn’s disease), immunosuppression in GVHD (Graft-versus-host disease), and multiple sclerosis [155,156,157,158,159,160]. The transfer of autologous free fat has found several applications especially in plastic surgery [161,162,163,164,165,166,167]. This includes: reconstruction and re-modelling of breast (either in post-oncologic or in de-novo resections) [162]; facial and hand rejuvenation [161]; facial re-modelling, following the appearance of the typical HIV (human immunodeficiency virus) lipodystrophy [163]; correction of asymmetry in the syndrome of Poland and Parry–Romberg syndrome [164]. It is believed that the presence of ASCs promotes the increase of angiogenesis with the consequent increase in capillary density. This promotes neovascularization and differentiation of adipocytes, preventing controlled death phenomena through the expression of VEGFA (vascular endothelial growth factor A) and IGF-1 (insulin-like growth factor 1) [167]. This application finds a good response also in craniofacial microsomia and in facial/cranial practices in general, although this requires close follow-up for further lipofilling applications [168,169]. Mailey et al. also showed positive results in the use of lipofilling mixed with ASCs in the maintenance of the symmetry, scars, and deformities, and a significant increase in skin improvement has been documented in patients undergoing this procedure [170]. In bone repair, expanded ASCs were used clinically in a maxillary flap implant with beta-tricalcium phosphate with the addition of bone protein 2. Eight months after implantation, it was reported that the flap had developed mature bone structures vascularization [171]. The calvaric defects of a 7-year-old girl with severe collapse were repaired using the adipose stem cells with fibrin glue, as confirmed by computed tomography. Currently, there are several ongoing clinical trials that use ASCs for bone repair [172,173].

The Table 2 reported some clinical trials in Phase I, II, and III with ASCs. The table revealed a widespread application of ASCs for the treatment of different types of disease.

### 3.3. Gene/Cell Therapy Approaches with Adipose Stem Cells

The gene therapy refers to innovative molecular procedures that offers the theoretical advantage of delivering a therapeutic gene in a genetically defective host cell (Figure 3). The biotechnology is highly efficient and effective and might provide a durable and potentially curative clinical benefit in a single treatment. This is guaranteed by the use of replication-defective viruses such as retro-viruses, adeno-associated, and lentivirus virus that transduces the therapeutic gene into the DNA of the recipient cells [174,175,176,177]. Here, the gene enters in the conventional transcription machinery that allows the production of the therapeutic protein. The combination of stem cells with gene delivery systems has significantly increased the therapeutic potential of gene therapy [137,138,139,178,179,180,181]. Good examples have been the clinical benefit of the transduction of autologous hematopoietic stem cells in patients with metabolic and storage disorders, immunodeficiencies, and hemoglobinopathies [127,128,182,183]. 

Many researcher groups have explored the potential of engineered ASCs. Below we report some examples in preclinical studies.

Morizono and co-authors demonstrated that the ASCs isolated from lipoaspirate can be transduced with lentiviral vectors with high efficiency and that the transgene expression was maintained after differentiation into adipogenic and osteogenic lineages [184].

The apoptosis of A375 melanoma cells was obtained by the co-culture of A375 cells with ASCs transduced with a lentiviral vector carrying the tumor necrosis factor-related-apoptosis-inducing-ligand (TRAIL)-cDNA [185]. Similarly, ASCs transduced with a lentiviral vector carrying the canine interferon β gene (cIFN-β) inhibited the growth of canine melanoma LMeC cells in an in vitro system. The effectiveness of this treatment was demonstrated in a melanoma mouse model (BALB/c nude mouse xenografts injected with LMeC cells). In this in vivo study, the combination of engineered ASCs with a low dose of the chemotherapeutic Cisplatin, significantly reduced the tumor volume in respect to the experimental control group [186]. In other work, the therapeutic potential of ASCs transduced with a lentiviral vector expressing the α-1 antitrypsin to reduce bone loss in mice was demonstrated. The transplantation of these engineered ASCs in an ovariectomized mouse model, significantly protected against induced bone loss [187].

Furthermore, human ASCs, transduced with a retroviral vector carrying the bifunctional fusion gene CD::uracil phosphoribosyltransferase, was used in combination with the chemotherapy drug 5-fluorocytosine (5-FC) to target colon cancer [188]. The same approach was successfully applied for the treatment of glioblastoma in a rat model [189].

Zhu et al. transplanted transduced ASCs with lentiviral vector encoding human hepatocyte growth factor (lens-hHGF: a growth factor with angiogenic, antifibrotic, and anti-inflammatory benefits) into the myocardium of an acute myocardial infarction rat model obtaining an improvement of the cardiac function. It was suggested that, in part, this result was a consequence of the capability of ASCs to differentiate into endothelial cells, resulting in increased blood flow and decreased fibrosis [190].

Finally, in a recent study, hASCs were transduced with the pCDH813A-1 lentiviral vector carrying the recombinant IL-23 decoy receptor (RIL-23R) gene to provide a useful approach for a basic research on cell-based gene therapy for autoimmune disorders [191].

### 3.4. Adipose Stem Cells and Tissue Engineering

The tissue engineering strategies are based on the restoration of damaged tissues/organs through the implantation of biohybrid systems that reproduce the architecture and the canonical functions of healthy tissues [4,192,193,194,195,196,197,198]. This is accomplished by combining stem cells and multifunctional biomaterials (Figure 3) [4,192,193,194,195,196,197,198,199].

The material used in tissue engineering applications must be biocompatible, they should not cause chronic body reactions and must be biodegradable through natural hydrolytic mechanisms, without the help of exogenous reagents (Table 3) [200,201]. Within the biohybrid system the biomaterial acts as a scaffold that mimics the three-dimensional structure of the tissue, generating a microenvironment as similar as possible to the extracellular cell matrix (ECM) that promotes and assists adhesion, proliferation, and differentiation process of stem cells [4,47,193,195,196,202]. Hence, the design of biomaterials with specific properties represents a valid approach for modulating and controlling the stem cell fate [194,196,197,203]. In fact, the modification of physical and chemical properties of biomaterials (e.g., dimensions, shape, mechanical properties, and surface structure) have been demonstrated to control the biological responses of the cells [4,47,192,193,197,198,202,204,205,206,207,208,209,210,211,212,213].

Biomaterials can be classified according to their polymeric composition in natural and biosynthetic systems (Table 3). Moreover, they are also distinguished in basic polymers and nanocomposites in which the characteristics and the structure of the polymers are modified by dispersion of different compounds or nanoparticles (see the summary Table 3 for a general overview).

The first interaction that takes place between the cells and biomaterial is cell adhesion. Therefore, surface properties of the scaffold become a key factor in governing the success of an engineered structure. The interaction of the cells to the surface is essential for determining the shape of the cell, for the maintenance of the correct proliferation rate, cell function, and tissue integrity [47,195,197,199,203,236]. The phenomenon that leads the cells to sense the different characteristics of the material and respond to this through the transduction of mechanical and physical stimuli into biochemical signals, is known as mechanotransduction [192,193,194,195,196,197,203]. Hence, the elucidation of the mechanotransduction axes is mandatory in order to shed light on complex biological phenomena such as stem cell determination processes, cell reprogramming pathways and behavior in the development phase [194,195,197,237]. The overall events drive the development of tissue engineering applications [4,47,237,238,239].

Adipose stem cells are widely used in combination with different types of biomaterials in order to achieve regeneration of various damaged tissues (see summary Table 4, for an overview). Here we documented some recent relevant successes scheduled according to the multipotential properties of ASCs (Table 4).

#### 3.4.1. Mesenchymal Tissues

**Adipogenic**. The natural function of ASCs to give rise to a functional adipose tissue might be improved by combination with different types of biomaterials (Table 4). A recent study has tested an extracellular matrix hydrogel, composed of soluble ECM obtained by the centrifugation of human adipose tissue and methylcellulose [240]. This led to the obtaining of a cell-free support system, that was injected subcutaneously in nude mice and activated the development of particular niches. The specific mechanical and biochemical characteristics induced the infiltration and differentiation of host cells, leading to the formation of a new and functional adipose tissue [240,241,242,304,305,306].

**Osteogenic**. During the last decade, many research groups have explored the potential of ASCs for the regeneration of bone tissue (Table 4). Osteogenic differentiation has been obtained by combining ASCs with natural, synthetic, and nanocomposite biomaterials (Table 4). Efforts have been made in identifying the best condition for osteogenic differentiation. For instance, ASCs could be directly induced toward endochondral ossification, that is the ability to generate bone with bone marrow, through an intermediate cartilage stage. The endochondral ossification, in fact, is a critical step in the regeneration of organs and in particular for the engineering of bone grafts [263]. The ectopic formation of bone tissue and bone marrow elements were also obtained by using the Adiscaf, a construct obtained from fractional lipoaspirate, after 3 weeks of culture [307]. In addition, the ASCs respond well to osteogenic differentiation through their interaction with bioactive glass nanoparticles and nanoparticles conjugated to strontium molecules. The results of Leite et al. showed that the products generated by the dissolved nanoparticles promoted the expression of key genes and proteins associated with osteogenic lineages in hASCs. This effect has been significantly improved by the presence of strontium, which induces osteogenic differentiation even without the use of osteogenic inducers [308].

Other studies conducted on biomaterials where the nanotopography was modified, allowed the collection of more information about the capacity of differentiation of ASCs towards the osteogenic lineage. Significant information was obtained by exploring the interaction of ASCs with different preparations of biodegradable polylactic acid nanoparticles (PLA) having different diameters. It was demonstrated that the osteogenic differentiation of hASCs can be guided by nanoparticles with a specific diameter of 200 nm [309]. It was also shown that the combination of hASCs with polycaprolactone (PCL) scaffolds allowed the anatomical and functional reconstruction of post-operative temporal bone defects after mastoidectomy (removal of infected mastoid bones) [256]. Interestingly, advances have recently been made in work on the shape-memory of polymers (polymeric intelligent materials that have the ability to return from a deformed state to their original shape due to the induction of an external stimulus). The procedure allows for producing programmable materials that are cytocompatible and capable of a non-invasive release for the patient. With this aim, it was investigated whether the osteogenic differentiation of ASCs can be maintained both during and after the activation of programmed shape changes of shape-memory polymeric scaffolds. Thus, ASCs were seeded in shape-memory polymer scaffolds modulated by body temperature and the results obtained supported the feasibility of using these innovative polymers for stem cell-based therapy intended for bone repair [310].

**Tendon**. The repair of tendon injuries has not yet brought satisfactory clinical results. Therefore, tissue engineering approaches by using ASCs, are today considered a promising alternative therapeutic strategy (Table 4). For instance, one approach is the manipulation of the intrinsic biochemical signals in the ECM of the native tendon and the biofactors present in the tissue of origin (decellularized ECM) combined with ASCs [311]. Further investigations have been carried out to study the mechanical properties of the native tissue in order to stimulate the differentiation towards tendon structures. To this end, the expression of activine (a member of the TGF-β superfamily responsible for the mechanical response of the stem cells [312]) in hASCs was induced by magnetic nanoparticles activated remotely through an oscillating magnetic bioreactor. This lead to the tenogenic differentiation and in turn a potential therapy for the regeneration of tendons [312].

**Cartilaginous**. Hyaluronic acid (HA) is a natural polymer that is necessary for the initial phase of chondrogenic differentiation [286]. Therefore, ASCs have been combined with hyaluronic acid to reproduce at best the characteristics of the chondrogenic niche. It was shown that the optimal molecular weight of hyaluronic acid useful to promote ASC chondrogenesis was 2000 kDa, thus providing and provide new tools for the regeneration of articular cartilage [286].

#### 3.4.2. Non-Mesenchymal Tissues

The evidence available for non-mesenchymal differentiation lineages is rather limited. In the following we report some recent results.

**Neural**. Several studies have demonstrated the capability of ASCs combined with proper biomaterials to regenerate the nerve tissues. For instance, Han and co-authors, combined multi-walled carbon nanotubes (MWCNTs) inserted in the polydimethylsiloxane polymer (PDMS/MWNT) (to ensure the achievement of greater mechanical strength and electroconductivity properties) and ASCs to generate neural differentiated cells. In this work, the ASCs and a mixture of glial growth factors were plated on PDMS/MWNTs and co-cultured with dorsal root ganglion neurons. The results obtained demonstrated a greater neuronal proliferation, a longer neurite outgrowth for neuron, and synergistic effects in the regeneration of peripheral nerves [271]. In another recent application, ASCs and Schwann cells seeded on hydrogel gelatin tubes were directly transplanted on the artificial sciatic lesion in mice promoting axon regeneration, myelin formation and denervation restoration [313].

**Cardiac**. ASCs are promising in the treatment of cardiac fibrosis due to their potential ability to block the differentiation of cardiac myofibroblasts, a determining factor for the appearance of cardiac fibrosis (Table 4) [273,274]. For this purpose, scaffolds consisting of polyacrylamide hydrogel coated in turn of collagen were synthesized. The pore size was adjusted without altering the matrix stiffness [274].

**Endothelial**. The presence of blood vessels is an essential element for the regeneration of tissues as they allow the exchange of oxygen and nutrients, an indispensable phenomenon to keep the tissues alive. The creation of new artificial vessels in vitro has been a challenge for a long time in tissue engineering. Promising results were obtained with the use of a nanofibrous structure of PCL/gelatin and a co-culture of ASCs [277]. Yet, combining ASCs and the elastin-like recombinamers (ELR)-based hydrogel, a viscoelastic material, caused the regeneration of blood vessels. In particular, the use of the ELR-based hydrogel allowed controlling the angiogenic events and the inflammatory process at the receiving site [279]. Finally, ASCs cultured on hydrogels and injected into the intramuscular environment of the hind limbs of a mouse model of human ischemia, caused the regeneration of blood vessels [314].

**Other tissues**. Skeletal muscle is characterized by a remarkable regenerative capacity. However, if extensive damage exceeds the self-regenerating capacity of the muscle it can lead to the formation of irreversible fibrosis and scarring with significant loss of function [315]. In this context, benefit was gained by ASCs transplanted with a collagen hydrogel into the crushed tibial muscle [297]. Good results have also been obtained from the use of polypyrrole-coated polymers (PPy) and electrical stimulation to differentiate ASCs to smooth muscle cells [298]. Finally, another important application is the capability of ASCs combined with polyvinyl alcohol (PVA) to control the inflammatory process related to obesity and in general with metabolic disorders, such as type 1 diabetes mellitus (DM1) [303].

## 4. Conclusions

Here, we have highlighted the relevance of ASCs as ex vivo model for the study of the biology of stem cells and their performance in regenerative medicine. The overall reports have described the current landscape of ASCs and emphasized that the future of ASCs is just beginning.

However, some critical aspects still need to be discussed as they could influence the effectiveness of ASC clinical applications.

One issue is related to the most common traditional method currently used for ASCs isolation. The method is based on the adhesion of ASCs to the culture plastic. This results in obtaining a heterogeneous population of stem and progenitor cells that could affect the differentiation rate [65]. Moreover, the absence of a universal isolation procedure increases the ASCs variability between donors making it difficult to standardize the clinical use of these stem cells [316].

The other issue is about the setting of ad hoc criteria for regenerative medicine applications. This includes: (i) the selection of the window of interning, (ii) the most effective site of transplantation, and (iii) the definition of the correct cell number to be transplanted [65].

No less relevant is the need to better elucidate the mechanisms that take place between transplanted ASCs and microenvironments. In this regard in vitro study although informative for clarifying selected signals cannot be informative for clinical implications. Similarly, data from clinical studies with ASCs can be difficult to interpret as they are derived from a wide spectrum of diseases, thereby limiting the stem cell’s clinical use and causing errors at the beginning of treatment [316].

## Figures and Tables

**Table 1:** Adult stem cell types. The table reports the characteristics of the main types of adult stem cells.

Adult Stem Cell Types	Embryonic Origin	Living Tissue	Multipotency	References
**Adipose Stem Cells**	Mesoderm	Adipose tissue and lipoaspirate	The adipose stem cells have the ability to differentiate toward different cells of several tissues: fat, bone, cartilage, skeletal, smooth and cardiac muscle, endothelium, hematopoietic, and liver and neuronal.	[38,39,40,41]
**Dental Stem Cells**	Mesoderm	Dental pulp	Dental stem cells may differentiate toward mesenchymal (osteoblasts, adipocytes, chondrocytes and myocytes) and non-mesenchymal (neuronal and endothelial cells, hepatocytes and melanocytes) tissue cells.	[42]
**Bone Marrow Mesenchymal Stem Cells**	Mesoderm	Bone-marrow	These stem cells could be induced to differentiate to the lineages of the mesenchymal tissues, including bone, cartilage, fat, tendon, muscle and medullary stroma. Recently, the ability of mesenchymal bone marrow stem cells to differentiate into cardiac, neuronal lineages, and hepatocyte-like cells has also been demonstrated.	[43,44,45,46,47]
**Hematopoietic Stem Cells**	Mesoderm	Peripheral blood, bone marrow, and umbilical cord blood	Hemopoietic stem cells give rise to both the myeloid (monocytes, macrophages, neutrophils, basophils, eosinophils, erythrocytes, megakaryocytes platelets), and lymphoid (T cells, B cells, and natural killer) cells lineages of blood cells.	[48,49,50]
**Epidermal Stem Cells**	Ectoderm	Epidermis and hair follicles	Epidermal stem cells can differentiate toward cells of at least three specialized structures: epidermis, hair follicles, and sebaceous glands.	[51,52,53]
**Neural Stem Cells**	Ectoderm	Adult brain and spinal cord	Neural stem cells give rise to differentiated neurons, astrocytes, and oligodendrocytes.	[54,55,56]
**Endothelial Stem Cells**	Endoderm	Endothelial intima of blood vessels, peripheral blood, bone marrow, and umbilical cord blood	Endothelial stem cells are implicated in functional blood vessels and lymphatic vascular systems formation.	[57,58]
**Umbilical Cord Mesenchymal Stem Cells**	Endoderm	Umbilical cord blood, umbilical vein subendothelium, and Wharton’s jelly	These stem cells may be differentiated in osteoblasts, chondrocytes, adipocytes, skeletal muscle cells, endothelial cells, hepatocytes, cardiomyocytes-like cells, and neurons.	[59,60,61]
**Joint/Synovium-Derived Mesenchymal Stem Cells**	Mesoderm	adult skeletal tissues synovium and bone marrow	These stem cells may be differentiated in chondrocytes, adipocytes, osteoblasts.	[62,63]

**Table 2:** Clinical trials. The table reports registered clinical trials on https://clinicaltrials.gov focused on adipose stem cells.

NCT Number	Title	Status	Conditions	Interventions	Sponsor/Collaborators	Phases
NCT03608579	Autologous Culture Expanded Adipose Derived MSCs for Treatment of Painful Hip OA	Recruiting	Osteoarthritis, Hip	Drug: Autologous Adipose Derived Mesenchymal Stromal Cells	Mayo Clinic	Phase I
NCT03570450	Regenerative Stem Cell Therapy for Stroke in Europe	Recruiting	Stroke	Drug: Adipose derived Stem Cell|Drug: placebo	University Hospital, Grenoble|European Commission H2020 program	Phase I
NCT03308565	Adipose Stem Cells for Traumatic Spinal Cord Injury	Recruiting	Spinal Cord Injuries|Paralysis	Biological: Autologous, Adipose derived Mesenchymal Stem Cells	Allan Dietz|Mayo Clinic	Phase I
NCT03279081	Adult Allogeneic Expanded Adipose-derived Stem Cells (eASC) for the Treatment of Complex Perianal Fistula(s) in Patients with Crohn’s Disease	Recruiting	Crohn’s Disease	Drug: Cx601|Other: Placebo	TiGenix S.A.U.|Cellerix	Phase III
NCT03268603	Intrathecal Autologous Adipose-derived Mesenchymal Stromal Cells for Amyotrophic Lateral Sclerosis (ALS)	Recruiting	ALS|Amyotrophic Lateral Sclerosis	Drug: Autologous Adipose-derived Mesenchymal Stromal Cells	Mayo Clinic|State of Minnesota Regenerative Medicine Minnesota	Phase II
NCT03171194	Pilot Trial of Mesenchymal Stem Cells for Systemic Lupus Erythematosus	Active, not recruiting	System; Lupus Erythematosus	Drug: Low Dose Mesenchymal Stem Cells (MSCs)	Medical University of South Carolina	Phase I
NCT03092284	Allogeneic Stem Cell Therapy in Heart Failure	Recruiting	Heart Failure	Biological: Cardiology Stem Cell Centre Adipose Stem Cell (CSCC_ASC)|Biological: Placebo	JKastrup|Rigshospitalet, Denmark	Phase II
NCT02952131	Use of Autologous, Adult Adipose-Derived Stem/Stromal Cells in Inflammatory Bowel Disease	Recruiting	Inflammatory Bowel Diseases	Procedure: Lipoaspiration|Procedure: AD-cSVF|Procedure: Normal Saline IV	Healeon Medical Inc|Terry, Glenn C., M.D.	Phase I|Phase II
NCT02904824	Injection Laryngoplasty Using Autologous Fat Enriched with Adipose Derived Regenerative Stem Cells (ADRC)	Completed	Vocal Cord Paralysis, Unilateral	Biological: adipose derived regenerative cells|Biological: centrifuged autologous fat	Hospital General Universitario Gregorio Marañon	Phase I|Phase II
NCT02808208	Autologous Adipose Derived Mesenchymal Stem Cells (AMSC) in Reducing Hemodialysis Arteriovenous Fistula Failure	Recruiting	End Stage Renal Disease (ESRD)|Vascular Access Complication	Biological: Adipose Derived Mesenchymal Stem Cells (AMSC)	Mayo Clinic	Phase I
NCT02741362	Safety and Efficacy of Adipose Derived Stem Cells in Refractory Rheumatoid Arthritis, Systemic Lupus Erythematosus or Sharp’s Syndrome	Terminated	Systemic Lupus Erythematosus|Rheumatoid Arthritis|Sharp’s Syndrome	Other: Intravenous injection of Stromal Vascular Fraction Cells (SVF) containing ADSCs|Other: Lipoaspiration	Arkansas Heart Hospital	Phase I
NCT02387723	CSCC_ASC Therapy in Patients with Severe Heart Failure	Completed	Heart Failure	Biological: Allogeneic adipose derived stem cells (CSCC_ASC)	JKastrup|Rigshospitalet, Denmark	Phase I
NCT02287974	Clinical Trial I/II Opened, Randomized and Controlled for the Study of the Use of Stem Cells Therapy in Insulinized Diabetic Patients Type 2 With Critical Ischemia in Lower Limbs (CLI): Study of the Needs of Insulin	Completed	Critical Limb Ischemia (CLI)	Drug: Stem cell infusion	Andalusian Initiative for Advanced Therapies—Fundación Pública Andaluza Progreso y Salud|Iniciativa Andaluza en Terapias Avanzadas	Phase I|Phase II
NCT02208713	Intramuscular Transplantation of Muscle Derived Stem Cell and Adipose Derived Mesenchymal Stem Cells in Patients with Facioscapulohumeral Dystrophy (FSHD)	Recruiting	Dystrophy	Biological: Intramuscular injection	Royan Institute	Phase I
NCT02161744	Safety, Tolerability and Preliminary Efficacy of Adipose Derive Stem Cells for Patients With COPD	Active, not recruiting	Chronic Obstructive Pulmonary Disease	Biological: ADSCs administration	Arkansas Heart Hospital	Phase I
NCT02068794	MV-NIS Infected Mesenchymal Stem Cells in Treating Patients with Recurrent Ovarian Cancer	Recruiting	Malignant Ovarian Brenner Tumor | Ovarian Serous Adenocarcinoma|Ovarian Transitional Cell Carcinoma| |Undifferentiated Ovarian Carcinoma	Other: Laboratory Biomarker Analysis|Procedure: Mesenchymal Stem Cell Transplantation|Biological: Oncolytic Measles Virus Encoding Thyroidal Sodium Iodide Symporter	Mayo Clinic|National Cancer Institute (NCI)	Phase I|Phase II
NCT02035085	19F Hot Spot MRI of Human Adipose-derived Stem Cells for Breast Reconstruction	Recruiting	Breast Cancer	Drug: CS-1000 labeled SVF cells	Johns Hopkins University|Cosmeticsurg.net	Phase I
NCT01828723	Safety Study of Antria Cell Preparation Process to Enhance Facial Fat Grafting with Adipose Derived Stem Cells	Completed	Lipoatrophy|Aging|Wrinkles	Biological: SVF	Antria	Phase I
NCT01678534	Reparative Therapy in Acute Ischemic Stroke with Allogenic Mesenchymal Stem Cells from Adipose Tissue, Safety Assessment, a Randomised, Double Blind Placebo Controlled Single Center Pilot Clinical Trial	Completed	Ischemic Stroke	Drug: Allogenic mesenchymal stem cells from adipose tissue|Drug: Placebo	Instituto de Investigación Hospital Universitario La Paz	Phase II
NCT01649687	Treatment of Cerebellar Ataxia with Mesenchymal Stem Cells	Completed	Cerebellar Ataxia	Biological: Allogeneic adult adipose-derived mesenchymal stem cells	National Yang Ming University	Phase I|Phase II
NCT01585857	ADIPOA—Clinical Study	Completed	Osteoarthritis	Biological: Autologous adipose derived stem cells administrated for intra-articular use	University Hospital, Montpellier	Phase I
NCT01532076	Effectiveness of Adipose Tissue Derived Mesenchymal Stem Cells as Osteogenic Component in Composite Grafts	Terminated	Osteoporotic Fractures	Procedure: Cellularized composite graft augmentation|Procedure: Acellular composite graft augmentation	University Hospital, Basel, Switzerland	Phase II
NCT01257776	Human Adipose Derived Mesenchymal Stem Cells for Critical Limb Ischemia (CLI) in Diabetic Patients	Completed	Critical Limb Ischemia (CLI)|Diabetes	Drug: Autologous adipose derived mesenchymal stem cells	Andalusian Initiative for Advanced Therapies—Fundación Pública Andaluza Progreso y Salud|Iniciativa Andaluza en Terapias Avanzadas	Phase I|Phase II
NCT01222039	Multicenter Clinical Trial for the Evaluation of Mesenchymal Stem Cells from Adipose Tissue in Patients with Chronic Graft Versus Host Disease	Completed	Graft Versus Host Disease|Chronic and Expanded Graft Versus Host Disease|Immune System Diseases	Other: Conventional treatment plus intravenous infusion of allogenic mesenchymal stem cells from adipose tissue	Andalusian Initiative for Advanced Therapies—Fundación Pública Andaluza Progreso y Salud|Iniciativa Andaluza en Terapias Avanzadas	Phase I|Phase II
NCT01157650	Treatment of Fistulous Crohn’s Disease by Implant of Autologous Mesenchymal Stem Cells Derived from Adipose Tissue	Completed	Crohn Disease	Other: Autologous mesenchymal stem cells	Clinica Universidad de Navarra, Universidad de Navarra	Phase I|Phase II
NCT01056471	Autologous Mesenchymal Stem Cells from Adipose Tissue in Patients with Secondary Progressive Multiple Sclerosis	Completed	Demyelinating Autoimmune Diseases, CNS|Autoimmune Diseases of the Nervous System	Other: Autologous mesenchymal stem cells from adipose tissue	Andalusian Initiative for Advanced Therapies—Fundación Pública Andaluza Progreso y Salud|Carlos III Health Institute	Phase I|Phase II
NCT00913289	Liver Regeneration Therapy Using Autologous Adipose Tissue Derived Stromal Cells	Terminated	Liver Cirrhosis	Biological: adipose tissue derived stromal cells	Kanazawa University	Phase I
NCT00442806	Randomized Clinical Trial of Adipose-Derived Stem Cells in the Treatment of Pts With ST-elevation Myocardial Infarction	Completed	Myocardial Infarction|Coronary Arteriosclerosis|Cardiovascular Disease|Coronary Disease	Drug: Injection of ADRC’s|Other: Injection of Placebo	Cytori Therapeutics	Phase I

Note: Only clinical trials which were in Phase I, Phase II, and/or Phase III were included.

**Table 3:** Biomaterials for tissue engineering. The table describes the characteristics of the most commune biomaterials used in tissue engineering.

Biomaterial	Description	References
**Natural Polymers**	Collagen, glycosaminoglycans, chitin, and chitosan have been used to repair a large number of defects on various organs such as nerves, skin, cartilage, and bones.	[214,215,216,217,218]
	Hyaluronic acid, used also as a gel, is widely used in the regeneration of soft tissues.	[219,220]
**Synthetic Polymers**	Such as: polyphenylene esters, polyanhydrides, and polyortoesters. This class of polymers includes polyglycolic (PGA), polylactic acid (PLA), and polycaprolactone (PCL) with different degradation times.	
	Poly-l-lactide (PLLA) produced by the polymerization of l, l-lactide is a biodegradable and bioactive thermoplastic aliphatic polyester derived from renewable resources such as corn starch (in the United States and Canada), cassava, fried potatoes or starch (especially in Asia), or sugar cane (in the rest of the world). PLLA has been employed for a wide range of tissue engineering purposes, such as bone, cartilage, tendon, neural, and vascular regeneration.	[221,222,223,224,225]
	PCL is synthesized by ring-opening polymerization of the cyclic monomer ε-caprolactone. It has been used since the 70s as a long-term resorbable sutures and implants.	[209,226]
**Nanocomposite Biomaterials**	By reinforcing the matrix with particles of nanometric dimensions, a clear improvement can be achieved in many physical and chemical properties of the scaffold, with a very low charge content, or even through the attribution of absent characteristics in the polymer as the thermal or electrical conductivity.	
	*NanoHydroxyapatite* (NHAP). Hydroxyapatite is the main part of the bone. Can be natural or synthetic. It is mainly used in bone regeneration.	[198,227]
	*Single walled carbon nanotubes* (SWCNTs) have diameter typically around 1 nm and length until some micrometer and are tubes made of a single sheet of graphene.	[228,229]
	*Multi-walled carbon nanotubes* (MWCNTs) are made from more graphene sheets, with diameter in the range of 10–20 nm.	[197,230]
	*Graphene*, *Graphene Oxide*. Graphene is a single layer of sp^2^-bonded carbon atoms in a hexagonal lattice. It is one of the most popular nanomaterials due to its excellent physical, electrical, and thermal properties. It is the strongest material ever measured. It has a Young’s modulus of 1 TPa, fracture toughness of 130 GPa, thermal conductivity of ≈10^3^ W·m^−1^·K^−1^, and electrical conductivity of ≈10^2^ S·cm^−1^. Graphene oxide is the oxidized form of graphene with hydroxyls, epoxides, diols, ketones, and carboxyl functional groups. The presence of oxygen on the edges and basal planes of graphene oxide increases its hydrophilicity.	[231,232,233]
	*Gold Nanoparticles (Au NPs)*. Nanoparticles of noble metals as gold have nanometer diameter and they show a very intense color, which is absent in the bulk material, due to the collective oscillation of the free conduction electrons.	[234]
**Surface Modifications**	*Plasma processing*. Surface modification techniques are mainly applied in order to modify the first part of the scaffold that come into contact with the biological entities.	[235,236]

**Table 4:** Adipose stem cells and tissue engineering applications. The table reports the most recent applications of tissue engineering with ASCs and biomaterials of different types.

Application	Biomaterials	Biological Effect	References
**Adipose Tissue**	Semicircular microfluidic channel	Quantification of responses and changes of stem cells and tumor cells to cutting streams at the interstitial level.	[237]
	Free hydrogel system based on a tissue-specific extracellular matrix	This application is a promising cell-free therapeutic approach for in situ adipose tissue regeneration.	[240]
	Hydrogel crosslinked by thiolated heparin and methacrylated hyaluronic acid	Induction and differentiation of ASCs towards the adipogenic lineage.	[241]
	Chitosan/β-glycerophosphate/collagen hybrid hydrogel	Generation of a larger number of adipocytes and vascularized adipose tissues.	[217]
	Biomimetic poly(ethylene)-glycol hydrogel with embedded peptides	It provides niches for stem cell differentiation and for soft tissue regeneration.	[242]
	Modified silica nanomaterials	Different functionalized silica nanoparticles surfaces generate different responses in ASC cultures.	[212]
	Large three-dimensional poly(glycerol sebacate)/poly(l-lactic acid) scaffolds	Adipose tissue engineering.	[243]
	Extracellular matrix from adipose tissue in electrospinning scaffold of polydioxanone	Adipose stem cell culture.	[244]
**Bone Tissue**	Thermo-gelling hydrogel scaffold containing platelet rich plasma and biphasic calcium phosphate	New bone formation at the site of the calvular bone defect in rabbits.	[245]
	Electrospun polyethersulfone/poly(vinyl) alcohol/platelet rich plasma nanofibrous scaffolds	Osteogenic differentiation for bone tissue engineering.	[246]
	3-D scaffolds with BMP-2 loaded core-shell fibers	Bone tissue engineering.	[247]
	Methacryloyl gelatin-based hydrogels	Interplay between osteogenesis and angiogenesis in vitro in bone tissue engineering application.	[248]
	The collagen fibroin-ELR (elastin-like recombinamer) blend	Improvement of the mechanical tensile properties of engineered scaffolds to promote bone differentiation.	[249]
	The heterogeneous deproteinized bone	Repair segmental bone defects and have a good potential to be used as graft material.	[250]
	Collagen containing resveratrol scaffolds	Provide useful biological signals that then stimulate the regeneration of the craniofacial tissue.	[251]
	3-D-graphene/arginine-glycine-aspartic acid peptide nano-island composite	Promote differentiation of ASCs to osteoblasts.	[252]
	Silk fibroin/chitosan thin film	Tissue engineering of bone, cartilage, adipose, and skin.	[253]
	The aligned-(NanoAligned™) and random-(NanoECM™) oriented PCL nanofiber-coated plates	PCL nanofiber is a suitable regenerative medicine application for canine patients in vivo.	[254]
	A composite hydrogel of collagen and supramolecular scaffold	Bone tissue engineering applications.	[255]
	PCL scaffolds and osteogenic differentiation medium	Anatomical and functional reconstruction of temporal bone defects following mastoidectomy.	[256]
	Different porosities of chitosan scaffolds	Osteogenic differentiation.	[257]
	Beta-tricalcium phosphate granules and supporting mesh	Cranial repair.	[258]
	Polypyrrole/chitosan scaffold with electrical stimulation	Bone defect therapy.	[259]
	Electrospun silk fibroin nanofibrous scaffolds with two-stage hydroxyapatite functionalization	Bone tissue engineering.	[260]
	The metal ion (Zn, Ag, and Cu) doped hydroxyapatite nano-coated surfaces	Osteogenic differentiation and cell adhesion capacity are higher on nanocoated surfaces that include Zn, Ag, and/or Cu metal ions.	[261]
	Polyethylenimine-mediated BMP-2 gene transfection in vitro	BMP-2 gene delivery and induction of osteogenic differentiation.	[262]
	Collagen sponges	Endochondral ossification.	[263]
	Methacrylated gellan-gum alone and combined with collagen type I hydrogels	Both hydrogel formulations induced ASCs towards osteogenic differentiation.	[264]
	Poly(l-lactic acid)/gelatin fibrous scaffold loaded with Simvastatin/β-Cyclodextrin-Modified Hydroxyapatite Inclusion Complex	Potential application in bone tissue engineering.	[265]
	Poly(dopamine) coating of 3-D printed poly(lactic acid) scaffolds	Promotes the osteogenic differentiation of ASCs.	[266]
**Peripheral Nerve/Neural Cells**	Graphene oxide and reduced graphene oxide mats	Neural differentiation of the ASCs and improvement of nerve repair.	[267]
	Cell transplantation of ASCs differentiated in to Schwann cell-like cells (SCLCs) in vitro	Repair sciatic nerve defects in rats and in general the peripheral nerve injury.	[268]
	Poly (ε-caprolactone) (PCL) and PCL/gelatin nanofibrous scaffolds coated with platelet-rich plasma (PRP)	Application in nerve tissue engineering.	[269]
	Polystyrene surface containing nanopore array-patterned substrate (NP)	NP lead to greater adhesion of ASCs on the substrate, growth of filopods, elongation of nuclei, and expression of specific neural markers compared to flat substrates.	[270]
	PDMS/MWNT sheets as a scaffold	Regeneration of peripheral nerves, similar to Schwann cells.	[271]
**Cardiomyocytes**	Decellularized bovine myocardial extracellular matrix-based films (dMEbF)	dMEbF mimics native ECM, but also induces cardiomyocyte-like cells differentiation.	[272]
	Cardiac extracellular matrix (cECM) hydrogel	Cardiac tissue engineering applications.	[273]
	Collagen coated polyacrylamide hydrogel substrates	Therapeutic use of ASCs in cardiac fibrosis therapy in future.	[274]
	3-D cell masses and self-assembling peptides	Promising application for therapeutic angiogenesis to treat myocardial infarction.	[275]
**Endothelial**	Poly(ε-caprolactone)/chitosan scaffold	Bladder tissue engineering.	[276]
	Polycaprolactone/gelatin nanofibrous scaffolds	Promote the generation of robust and functional microvasal structures that could be valuable for regeneration of blood vessels.	[277]
	PEG hydrogel containing calcium-releasing particles	Vascular stabilization and revascularization of ischemic tissues.	[278]
	Elastin-like recombinamer-based hydrogel	Improve the successful integration of engineered substitutes in angiogenic and inflammation process.	[279]
	Surface-modified bioresorbable electrospun scaffolds from blends of poly(l-lactic acid) and segmented polyurethane	Vascular tissue engineering due to its biomimetic behaviors and its ability to avoid thrombus formation and provide antimicrobial characteristics.	[280]
	Hydrogel from gelatin-tyramine and chitosan-4-hydroxylphenyl acetamide	Vehicle for delivering signals to cells and growth factors by supporting the vascularization process in tissue engineering applications.	[281]
	Polyurethane urea (PEUU)	Engineered vascular grafts.	[282]
	Polycaprolactone (PCL)-gelatin mesh	Smooth muscle cells and endothelial cells for the bioengineering of small-diameter blood vessels.	[283]
**Tendon**	Magnetic cell sheet	Tendon therapies.	[284]
	Poly(l/d)lactide 96l/4d copolymer filament scaffolds in tenogenic medium compared to foamed poly(l-lactide-*co*-ε-caprolactone) 70L/30CL scaffolds	Tendon injury treatments.	[285]
**Cartilaginous**	Hyaluronan (HA)	Regeneration of hASC-mediated cartilage in chondral defects and cartilage joint.	[286]
	Cartilage extracellular matrix-derived particles	Alternative method for the culture of chondrocytes and stem cell differentiation; promising strategy for the construction of cartilage microtexes and repair of articular cartilage in vivo.	[287]
	Hyaluronic acid (HA)-modified thermoresponsive poly(*N*-isopropylacrylamide) hydrogels	Chondrogenesis and repair of the articular cartilage.	[288]
	3-d-photocrosslinkable methacrylated gelatin hydrogels	The hydrogel pre-loaded with TGF-β3 allows improvement of healing of the radial meniscus in an in vitro meniscal repair model.	[289]
	Alginate microbeads	Regeneration of auricular cartilage.	[290]
	Herbal extracts icariin and transforming growth factor β3 (TGFβ3) were added in fibrin-cell constructions	Chondrogenesis and together with TGF3 it could decrease its hypertrophic effects.	[291]
	Gelatin/chondroitin-6-sulfate/hyaluronan/chitosan highly elastic cryogels	Cartilage engineering.	[292]
	Polycaprolatone and with freeze thaw freeze scaffolds	Transition mechanism from chondroblast to chondrocyte.	[293]
	Polyvinyl alcohol/chitosan scaffold	Regeneration of the tear meniscus lesion.	[294]
	Composite gel based on collagen/hyaluronic acid	Chondrogenic differentiation in a dose-dependent manner.	[295]
	Poly-ε-caprolactone films	Implantation at sites of defects as a possible treatment of cartilage defects.	[296]
**Other Tissues**	Collagen hydrogel	Regeneration of muscle tissue.	[297]
	Polypyrrole-coated polymer scaffolds and electrical stimulation	Vascular smooth muscle regeneration.	[298]
	Poly(l-lactide)/Poly(e-caprolactone) electrospinning nano-scaffold	Myogenic proliferation and differentiation of ASCs.	[299]
	Acellular human amniotic membrane (HAM)	Application of cell-based skin substitutes.	[300]
	β-tricalcium phosphate (betaTCP)	Reconstruction of cranial defects.	[258]
	Injectable hydrogels	Immunomodulatory effects, angiogenic stimulation angiogenic.	[301]
	Polyethylene glycol-fibrin hydrogels	Improve wound healing and minimize donor or scar sites.	[302]
	Autologous growth factors and nanofibrous scaffolds	Pancreatic tissue engineering applications and beta cell replacement therapies in type 1 diabetes mellitus.	[303]
